# Evidence for a purifying selection acting on the β-lactamase locus in epidemic clones of methicillin-resistant *Staphylococcus aureus*

**DOI:** 10.1186/1471-2180-11-76

**Published:** 2011-04-15

**Authors:** Catarina Milheiriço, Ana Portelinha, Ludwig Krippahl, Hermínia de Lencastre, Duarte C Oliveira

**Affiliations:** 1 Laboratory of Molecular Genetics, Instituto de Tecnologia Química e Biológica, Universidade Nova de Lisboa (ITQB/UNL), Oeiras, Portugal; 2 CREM, Department of Life Sciences, Faculdade de Ciências e Tecnologia, Universidade Nova de Lisboa (FCT/UNL), Caparica, Portugal; 3 CENTRIA, Department of Informatics, Faculdade de Ciências e Tecnologia, Universidade Nova de Lisboa; 4 Laboratory of Microbiology, The Rockefeller University, New York, NY, USA

**Keywords:** β-lactamase, β-lactam resistance, allelic variation, MSSA, MRSA, *mecA *stabilization

## Abstract

**Background:**

The β-lactamase (*bla*) locus, which confers resistance to penicillins only, may control the transcription of *mecA*, the central element of methicillin resistance, which is embedded in a polymorphic heterelogous chromosomal cassette (the SCC*mec *element). In order to assess the eventual correlation between *bla *allotypes and genetic lineages, SCC*mec *types and/or β-lactam resistance phenotypes, the allelic variation on the *bla *locus was evaluated in a representative collection of 54 international epidemic methicillin-resistant *Staphylococcus aureus *(MRSA) clinical strains and, for comparative purposes, also in 24 diverse methicillin-susceptible *S. aureus *(MSSA) strains.

**Results:**

Internal fragments of *blaZ *(the β-lactamase structural gene) were sequenced for all strains. A subset of strains, representative of *blaZ *allotypes, was further characterized by sequencing of internal fragments of the *blaZ *transcriptional regulators, *blaI *and *blaR1*. Thirteen allotypes for *blaZ*, nine for *blaI *and 12 for *blaR1 *were found. In a total of 121 unique single-nucleotide polymorphisms (SNP) detected, no frameshift mutations were identified and only one nonsense mutation within *blaZ *was found in a MRSA strain. On average, *blaZ *alleles were more polymorphic among MSSA than in MRSA (14.7 *vs *11.4 SNP/allele). Overall, *blaR1 *was the most polymorphic gene with an average of 24.8 SNP/allele. No correlation could be established between *bla *allotypes and genetic lineages, SCC*mec *types and/or β-lactam resistance phenotypes. In order to estimate the selection pressure acting on the *bla *locus, the average dN/dS values were computed. In the three genes and in both collections dN/dS ratios were significantly below 1.

**Conclusions:**

The data strongly suggests the existence of a purifying selection to maintain the *bla *locus fully functional even on MRSA strains. Although, this is in agreement with the notion that in most clinical MRSA strains *mecA *gene is under the control of the *bla *regulatory genes, these findings also suggest that the apparently redundant function of *blaZ *gene for the MRSA resistant phenotype is still important for these strains. In addition, the data shows that the sensor-inducer *blaR1 *is the primary target for the accumulation of mutations in the *bla *locus, presumably to modulate the response to the presence of β-lactam antibiotic.

## Background

*Staphylococcus aureus *is a leading cause of nosocomial infections and has recently emerged as a community acquired pathogen [1-3]. *S. aureus *is also a paradigm of adaptive power to antimicrobial chemotherapy, able to develop resistance to virtually all classes of antibiotics [4].The acquisition of resistance to β-lactam antibiotics is particularly relevant in clinical terms. Although β-lactams (i.e. penicillin G) were the first class of large-spectrum antibiotics to be introduced into clinical practice, they are still the most widely used due to their high effectiveness, low cost, ease of delivery and minimal side effects [5].

In response to β-lactam chemotherapy, *S. aureus *has sequentially acquired two resistance genes: first *blaZ*, which codes for a β-lactamase and confers resistance to penicillins only, and then *mecA*, which codes for an extra penicillin-binding protein (PBP2a) with reduced affinity for virtually all β-lactams [6,7]. The transcription of both resistance genes may be controlled by homologous two-component systems consisting on a sensor-inducer (BlaR1 and MecR1) and a repressor (BlaI and MecI). Interestingly, in spite of the cross-resistance to virtually all β-lactams provided by *mecA*, the great majority (> 95%) of contemporary MRSA are still positive for the β-lactamase locus [8]. Moreover, the regulators of *blaZ*, BlaR1 and BlaI, can efficiently induce *mecA *transcription and, do it faster than the "natural" *mecA *regulators, MecR1 and MecI [9,10]. In addition, since many MRSA strains do not have functional *mecI-mecR1 *genes due to polymorphisms in the *mecA *regulatory region [11], the *mecA *transcription is presumably under the control of the *blaI-blaR1 *genes only. In line with these observations, the presence of the *blaZ *locus has been shown to promote *mecA *acquisition and stabilization [12,13].

In *S. aureus*, the β-lactamase genes may be located in a plasmid or mobilized into the chromosome by transposon Tn552 [14]. In contrast to the diversity of β-lactamase genes found in gram-negative bacteria, all staphylococcal enzymes studied so far are molecular class A serine β-lactamases placed in functional group 2a [8]. The mature form of the enzyme has a molecular mass of 30 kDa, contains 257 amino acids, and is secreted extracellularly [15]. In 1965, Richmond proposed the subdivision of staphylococcal β-lactamases in four serotypes [16], but the structural basis of the distinction between types is still uncertain and no clear relationship between sequence and serotype was found [17]. Interestingly, serotypes were shown to have specific geographic distributions [8], which may suggest a relationship between *bla-*type and genetic lineage. Recently, Olsen *et al *have studied the allelic variation of the *blaZ *gene among several staphylococcal species and 11 BlaZ protein types were identified [14]. The multiple-sequence alignment of those sequence types suggest a separate evolution for plasmid- and chromosomally-encoded *blaZ *and a very low frequency for exchange of the β-lactamase locus between strains and species.

In evolutionary terms, MRSA may be regarded as a recent sub-branch of the *S. aureus *population which has acquired the heterelogous chromosomal cassette containing the *mecA *gene - the SCC*mec *element [18]. Molecular epidemiology studies on large collections of MRSA isolates have clearly shown that MRSA has a strong clonal structure and that very few lineages, defined by specific macro-restriction patterns of chromosomal DNA and/or multi-locus sequence types, account for the great proportion of MRSA infections worldwide [19,20]. The clonal structure of MRSA population may result from a "host barrier" for the *mecA *acquisition, which restricts the number of acquisitions to few more permissive lineages [13,21] and/or from the clonal expansion of previously highly epidemic (MSSA) lineages, which have acquired the *mecA *gene. Recent data based on comparative genomics of MRSA lineages [22-24] supports both mechanisms as it seems that, within the same genetic (epidemic) lineage, SCC*mec *acquisitions may occur continuously at the local level.

In spite of the several lines of evidence suggesting an important role of the *bla *locus in the acquisition, stabilization and regulation of the *mecA *gene, the variability of *bla *genes at the sequence level has never been evaluated among pandemic MRSA lineages. The present study was conducted in order to evaluate the allelic variability of β-lactamase locus in a representative collection of internationally epidemic MRSA clones and also, for comparative purposes, in a diverse collection of methicillin-susceptible *S. aureus *strains (MSSA), in an attempt to make evolutionary correlations between β-lactamase allotypes and β-lactam resistance phenotypes (i.e. MRSA *vs *MSSA), SCC*mec *types and/or genetic lineages.

## Methods

### Strain collection

*S. aureus *strains used in the present study are listed in Tables 1 (MRSA) and 2 (MSSA). All strains have been previously assigned to genetic lineages by Pulse-field gel electrophoresis (PFGE), multi-locus sequence typing (MLST) and protein A sequence typing (*spa *typing). MRSA strains have been additionally characterized in terms of their SCC*mec *types. The presence of a functional β-lactamase locus was confirmed by nitrocefin disks (Sigma) for all strains, in the presence and absence of an inducer (oxacillin at 0.05 mg/L).

**Table 1:** Characteristics and *bla *locus allotypes of the MRSA strains used in this study

**Clonal complex ^a)^**	MLST (ST)	SCC*mec *type	Strain	Isolation origin	Isolation year	*bla *locus alleles			**Ref**.
							
						*blaZ*	*blaI*	*blaR1*	
	247	I	E2125	Denmark	1964	1	ND	ND	[30,31]
	247	IA	HPV107	Portugal	1992	1	ND	ND	[30,32]
	247	IA	BK1953	USA	1995	1	ND	ND	[30,33]
	250	I	COL	UK	1965	-			[30]
	250	I	BK793	Egypt	1961	1	1	1	[30,34]
	250	IA	PER34	Spain	1989	1	ND	ND	[30,35]
	239	III	ANS46	Australia	1982	1	1	1	[30,36]
	239	IIIA	HU25	Brazil	1993	1	ND	ND	[30,37]
	239	IIIA	HUSA304	Hungary	1993	1	ND	ND	[30,38]
8	239	IIIA	BK2421	USA	1996	1	ND	ND	[30,34]
	8	IVa	USA300	USA	1995-2003	8	4	9	[39,40]
	8	IVa	USA500	USA	1995-2003	1	ND	ND	[39,40]
	8	IVd	BK2529	USA	1996	1	ND	ND	[30,34,39]
	8	IVd	BargII17	USA	1996	1	ND	ND	[30,39,41]
	8	IVE	AR43/3330.1	Ireland	1988-2002	1	1	1	[42]
	8	IVh	GRE120	Greece	1993	1	ND	ND	[39,43]
	72	IVa	USA700	USA	1995-2003	9	-	4	[39,40]
	254	IVh	HAR36	Greece	2002	1	1	1	[39,44]
	770	IVb	8/6-3P	Chicago	1996	3	3	6	[45]

	5	I	HAR21	Finland	2002	1	ND	ND	[44,46]
	5	I-VAR	PL72	Poland	1991	1	ND	ND	[30,47]
	5	II	N315	Japan	1982	8	4	9	[18]
	5	II	JP1	Japan	1987	8	4	9	[30,48]
	5	II	BK2464	USA	1996	4	6	2	[30,49]
	5	II	USA100	USA	1995-2003	3	3	6	[40,46]
	5	IVa	BM18	USA	1989	4	6	2	[30,39,50]
	5	IVa	FFP311	Portugal	1996	11	1	7	[39,51]
5	5	IVa	HSA49	Portugal	1993	11	ND	ND	[39,51]
	5	IVa	HSA74	Portugal	1993	5	3	3	[39,51]
	5	IVc	Q2314	Dallas	1996	3	ND	ND	[52]
	5	IVc	USA800	USA	1995-2003	1	ND	ND	[39,40]
	5	IVc	ARG9	Argentina	1996	11	7	7	[39,51]
	5	IVd	JCSC4469	Japan	1982	1	1	1	[53]
	5	IVg	M03-68	Korea	2003	3	ND	ND	[54]
	5	IV_NT_	COB3	Colombia	1996	6	5	10	[30,39,55]
	5	VI	HDE288	Portugal	1996	10	-	5	[51,56]
	5	VI	HUC136	Portugal	1995	10	-	5	[51,56]
	228	I	HAR40	Belgium	1995	2	3	1	[44,46]

	30	IVc	DEN2946	Denmark	2001	1	1	1	[39,57]
30	30	IVc	DEN2294	Denmark	2001	1	ND	ND	[39,57]
	36	II	USA200	USA	1995-2003	1	1	1	[40,46]
	36	II	HAR24	Finland	2002	1	ND	ND	[46,47]

	22	IVh	HAR22	Finland	2002	9	-	4	[39,44]
22	22	IVh	HGSA146	Portugal	2003	9	ND	ND	[39,58]
	22	IVh	HGSA163	Portugal	2003	9	ND	ND	[39,58]

	45	II	USA600	USA	1995-2003	7	4	9	[40,46]
45	45	IVa	HAR38	Belgium	1995	6	2	8	[39,44]
	45	V	WIS	Australia	1995	8	ND	ND	[59]
	256	IVa	CA05	Chicago	1999	8	4	9	[45]

1	1	IVa	MW2	USA	1998	6	2	10	[60]
	1	IVa	USA400	USA	1995-2003	6	2	10	[39,40]

80	80	IVc	DEN2949	Denmark	2001	5	3	3	[39,57]
	80	IVc	DEN114	Denmark	2001	5	1	3	[39,57]

Singleton	377	V	HT0184	Greece	2005	6	2	10	[61]
	377	V	HT0826	France	2003	6	ND	ND	[61]

a) Clonal complexes were determined using the E-burst software http://saureus.mlst.net/, last accessed on June 04, 2009.

ND, not determined, -, negative PCR amplification.

**Table 2:** Characteristics and *bla *locus allotypes of MSSA strains used in this study^a)^

**Clonal complex ^b)^**	MLST (ST)	PFGE type	Strain	Origin	Isolation date	*bla *locus alleles		
						
						*blaZ*	*blaI*	*blaR1*
	1	G	IPOP38	Portugal	2001	6	2	10
1	188	L	IPOP58	Portugal	2001	6	2	10
	573	M	HSJ109	Portugal	1995	6	2	10

5	5	C	HSA29	Portugal	1992-1993	11	4	7
	5	C	IPOP41	Portugal	2001	6	3	6

8	8	J	IPOP65	Portugal	2001	8	2	ND
	615	E	IPOP32	Portugal	2001	9	1	4

9	9	D	HSJ122	Portugal	1995	12	1	12

10	10	Q	DCC300	Portugal	1996-1997	9	1	5

12	12	X	HSJ130	Portugal	1995	3	3	6
	12	X	Draftees728	Portugal	1996-1997	1	1	1

15	15	K	HSA9	Portugal	1992-1993	6	9	ND

20	20	N	HSA47	Portugal	1992-1993	6	8	11

22	22	T	Draftees721	Portugal	1996-1997	6	3	5

25	25	S	HSA76	Portugal	1992-1993	1	1	1

	30	A	IPOP37	Portugal	2001	13	1	1
30	34	B	IPOP24	Portugal	2001	6	ND	ND
	34	B	IPOP34	Portugal	2001	1	1	ND
	NA	B	IPOP26	Portugal	2001	1	ND	ND

45	45	H	HSA19	Portugal	1992-1993	6	2	10
	45	H	IPOP56	Portugal	2001	6	ND	ND

97	97	P	IPOP50	Portugal	2001	6	ND	ND

121	121	F	IPOP44	Portugal	2001	10	1	5

Singleton	580	R	DCC1185	Portugal	1996-1997	1	1	1

a) MSSA strains have been previously characterized by PFGE and MLST[62].

b) Clonal complexes were determined using the E-burst software http://saureus.mlst.net/eburst/database.asp, last accessed on June 04, 2009.

NA, not available; ND, not determined.

### Media and growth conditions

Strains were grown overnight at 37°C on tryptic soy agar or tryptic soy broth under aerobic conditions.

### DNA isolation

Total DNA was prepared using the Wizard genomic DNA preparation kit (Promega, Madison, WI, USA), according to the manufacturer's recommendations, except for the addition of lysostaphin at 0.5 mg/mL and RNase at 0.3 mg/mL for the lysis step.

### DNA amplification and sequencing

The allelic variation on the β-lactamase locus was evaluated by sequencing internal fragments of *blaZ *and its transcriptional regulators, *blaI *and *blaR1*, amplified by PCR. Based on the available sequence at GenBank (accession number: X52734) for Tn*552 *of *S. aureus*, three pairs of primers were designed as follows (5' → 3'): blaZ F1, GAT AAG AGA TTT GCC TAT GC; blaZ R1, GCA TAT GTT ATT GCT TGA CC; blaI F1, GCA AGT TGA AAT ATC TAT GG; blaI R1, GAA AGG ATC CAT TTT CTG TAC ACT CTC ATC; blaR1 F1, CAT GAC AAT GAA GTA GAA GC; and blaR1 R1, CTT ATG ATT CCA TGA CAT ACG. The predicted amplicon sizes were 533 bp for *blaZ*, 484 bp for *blaI *and 537 bp for *blaR1*. PCR was performed in a T1 Thermocicler (Biometra) with the following conditions: 94°C for 4 min; 30 cycles of 94°C for 30 s, 55°C for 30 s and 72°C for 1 min; and a final extension at 72°C for 10 min. In each reaction (final volume of 50 μL), 5 ng of chromosomal template, 2.5 U of GoTaq flexi DNA polymerase (Promega), 1× Colorless GoTaq flexi buffer (Promega), 2.5 mM MgCl2 (Promega), 40 μM of each deoxynucleoside triphosphate (dNTPs mixture, Bioron) and 20 pmol of the forward and reverse primers were used. The amplified fragments were purified using a mix of Exonuclease and SAP enzymes. Sequencing of both strands was performed by Macrogen http://www.macrogen.com or STAB Vida http://www.stabvida.com.

### DNA sequences analysis and phylogenetic tree reconstruction

DNA sequencing raw data analysis and multi-sequence alignments were performed using the DNA Star software package (Lasergene). For the multi-sequence alignments, the Clustal W algorithm was used. In order to maximize sequence reads, raw sequences for *blaZ *and *blaR1 *were trimmed immediately after the primer sequences keeping the reading frame. As the reverse primer for *blaI *(BlaI R1) is located outside of the coding region, the 3' end of the sequence was trimmed at the end of the coding region. For each gene, allotypes were defined taking as reference the extant sequences of the *bla *locus of Tn*552*, which were assigned to allotype 1.

Phylogenetic and molecular evolutionary analyses were conducted using *MEGA *version 4 [25] and the resultant phylogenetic trees were obtained using the neighbour-joining (NJ) method with bootstrap analysis using 1000 replicates. In order to evaluate the diversity of the *bla *locus, the Simpson's indexes of diversity (SID) were calculated [26,27] for each locus using the online tool available at http://www.comparingpartitions.info. To estimate selection pressure acting on the *bla *locus, we computed the dN/dS ratios for the three genes. The dN/dS ratios were computed for all pairs of alleles with more than 1% substitutions, in order to give an estimate of the divergence of the alleles while excluding those pairs that, being too similar, would give anomalous dN/dS ratios. The dN/dS ratios were computed by Model Averaging, as described in [28] and implemented in the KaKs_Calculator application [29]. This approach fits a set of models by maximum likelihood and then computes the weighted average of the models using a second-order Akaike Information Criterion (AICC).

### Nucleotide sequence accession numbers

All nucleotide sequences determined in this study were deposited in Genbank under accession numbers GQ980053-GQ980139 (*blaZ *alleles), GQ980140-GQ980187 (*blaI *alleles) and GQ980188-GQ980236 (*blaR1 *alleles).

## Results

The allelic variation in the β-lactamase locus (*bla*) was evaluated by sequencing internal fragments of *blaZ*, *blaI *and *blaR1 *genes in a representative collection of international epidemic MRSA clones and also, for comparative purposes, in a diverse collection of MSSA strains.

### *blaZ *allelic variability

**Figure 1:**
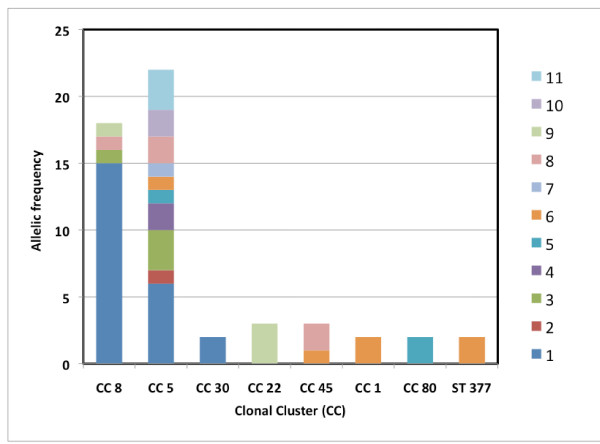
***blaZ *allotype frequency per MRSA lineage as defined by MLST clonal cluster**.

**Figure 2:**
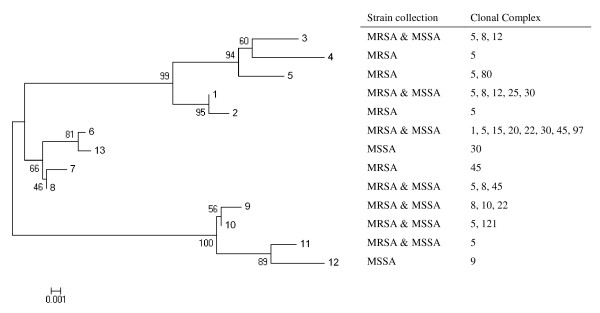
**Cluster tree of *blaZ *gene allotypes found in the MRSA and MSSA collections**. The tree was constructed with the neighbor-joining (NJ) method. In each branch is shown the corresponding bootstrap NJ values, taken over 1000 replicates, which assigns confidence values for the groupings in the tree. For each allele, it is indicated the collection(s) (MRSA or MSSA) and genetic lineage (clonal cluster) where it was found.

Thirteen different *blaZ *allotypes were identified within our collection, which comprised 54 MRSA and 24 MSSA (Tables 1 and 2, respectively). Although seven alleles were common to MRSA and MSSA strains, we found four alleles present in MRSA strains only and two present in MSSA strains only. Moreover, the relative frequencies of each allele were different among MRSA and MSSA strains (Table 3); for instance, *blaZ *allotype 1 was dominant in MRSA strains accounting for 43% (23 out of 54) of the isolates whereas in MSSA it accounted for 21% (5 out of 24) of the isolates, and *blaZ *allotype 6 was present in 11% (6 out 54) of MRSA but was dominant among MSSA accounting for 46% (11 out 24) of the isolates. The diversity of *blaZ *gene as measured by the Simpson index of diversity (SID) was higher for the MRSA collection than for MSSA, although not statistically significant due to the partial overlapping of the confidence intervals (SID = 79.18, 95%CI 69.6-88.8 *vs *SID = 76.09, 95%CI 61.3-90.9, respectively) - see Table 4. Within the length of *blaZ *region analyzed (492 nucleotides), we detected 43 unique single-nucleotide polymorphisms (SNP) and on average, each *blaZ *allele has 12.4 SNP comparing to the prototype *blaZ *sequence of Tn*552 *(allele 1) - see Tables 3 and 4. Overall, *blaZ *alleles were more variable in MSSA than in MRSA (14.7 and 11.4 SNP/allele, respectively). As illustrated by the allelic frequency distribution per MRSA lineage (Figure 1) or the cluster tree of the thirteen *blaZ *alleles found in our collections (Figure 2), there is no clustering according to genetic lineages, as defined by MLST sequence type and SCC*mec *type, or MSSA/MRSA phenotype; i.e. the same allele could be detected in different genetic lineages or among MRSA and MSSA, and the same lineage could be characterized by several alleles. In addition, there was also no clear clustering of *blaZ *allotypes according to geographic origin or isolation date of the MRSA isolates (see Table 1).

**Table 3:** Characteristics of *bla *locus alleles

Gene	Allele No.	Frequency		**SNP**^**c)**^	Amino acid substitutions			
				
		**MRSA**^**a)**^	**MSSA**^**b)**^		Silent	Conservative	Missense	Nonsense
	1	0.43	0.21	0	0	0	0	0
	2	0.02	0	1	0	0	1	1
	3	0.07	0.04	9	4	2	2	0
	4	0.04	0	9	4	2	3	0
	5	0.06	0	7	2	2	3	0
	6	0.11	0.46	13	8	2	3	0
***blaZ***	7	0.02	0	12	6	2	4	0
	8	0.10	0.04	11	6	2	3	0
	9	0.07	0.08	20	9	2	7	0
	10	0.04	0.04	19	8	2	7	0
	11	0.06	0.04	24	11	3	8	0
	12	0	0.04	24	11	2	8	0
	13	0	0.04	12	7	2	3	0

	1	0.33	0.45	0	0	0	0	0
	2	0.15	0.25	6	5	0	1	0
	3	0.19	0.15	1	0	0	1	0
	4	0.19	0.05	4	3	0	1	0
***blaI***	5	0.04	0	7	5	0	2	0
	6	0.07	0	4	3	0	1	0
	7	0.04	0	5	4	0	1	0
	8	0	0.05	3	1	1	1	0
	9	0	0.05	1	0	0	1	0

	1	0.26	0.24	0	0	0	0	0
	2	0.07	0	19	9	4	6	0
	3	0.10	0	18	7	4	6	0
	4	0.07	0.06	35	15	9	10	0
	5	0.07	0.18	35	15	7	11	0
	6	0.07	0.12	17	6	4	6	0
***blaR1***	7	0.07	0.06	24	10	7	7	0
	8	0.03	0	33	12	6	12	0
	9	0.16	0	31	11	6	11	0
	10	0.13	0.24	32	12	6	11	0
	11	0	0.06	20	9	5	7	0
	12	0	0.06	34	16	6	10	0

a) The total number of MRSA strains whose *blaZ*, *blaI *and *blaR1 *genes were analyzed is 54, 27 and 31, respectively.

b) The total number of MSSA strains whose *blaZ*, *blaI *and *blaR1 *genes were analyzed is 24, 20 and 17, respectively.

c) For each allele, the SNP were counted taking as reference Tn*552 bla *sequences (allele 1).

**Table 4:** Comparative analysis of the allelic variation in *bla *locus for MRSA and MSSA strains

		No. isolates	No. alleles	Simpson's		Unique SNP	SNP/allele (average)	Mutations per allele (average)				dN/dS	
							
				ID	CI (95%)			Silent	**Conserv**.	Missense	Nonsense	Average	**St. dev**.
	***blaZ***	54	11	79.2	69.6-88.8	41	11.4	5.3	1.7	3.7	0.1	0.21	0.11
**MRSA**	***blaI***	27	7	82.1	74.6-89.5	10	3.9	2.9	0	1.0	0	0.11	0.05
	***blaR1***	31	10	88.8	83.2-94.4	60	24.4	9.7	5.3	8.0	0	0.24	0.11

	***blaZ***	24	9	76.1	61.3-90.9	35	14.7	7.1	1.9	4.6	0	0.17	0.04
**MSSA**	***blaI***	20	6	74.2	60.5-87.9	9	2.5	1.5	0.2	0.8	0	0.08	0.03
	***blaR1***	17	8	88.2	81.2-95.3	61	24.6	10.4	5.5	7.8	0	0.24	0.10

	***blaZ***	78	13	81.1	75.0-87.3	43	12.4	5.8	1.8	4.0	0.1	0.20	0.10
**All**	***blaI***	47	9	78.4	71.0-85.9	13	3.4	2.3	0.1	1.0	0	0.10	0.04
	***blaR1***	48	12	88.5	84.0-93.0	65	24.8	10.2	5.3	8.1	0	0.25	0.10

ID, index of diversity; CI, confidence interval; SNP, single-nucleotide polymorphism; Conserv., conservative; St. dev., standard deviation

The BlaZ variability in the MRSA and MSSA strains at the protein level was evaluated by comparison of the deduced amino acid sequence of all alleles against the deduced amino acid sequence for the BlaZ of Tn*552*. Overall, the deduced amino acid sequences of *blaZ *alleles from the MRSA and MSSA strains revealed on average 5.8 silent mutations, 1.8 conservative missense mutations and 4 non-conservative missense mutations per allotype (see Tables 3 and 4). For MRSA strain HAR40, a nonsense mutation at Gln76 was detected which presumably originates a non-functional truncated BlaZ protein. As this strain was positive for the nitrocefin test, the DNA extraction and the *blaZ *sequencing were repeated and the nonsense mutation was confirmed. No frameshift mutations were found in *blaZ *allotypes.

### Allelic variability of *blaZ *regulatory genes

Based on the *blaZ *variability analysis, we selected 51 representative strains to further characterize the variability in the *blaZ *regulatory genes, *blaI *and *blaR1*. Some of these strains failed in the amplification of one of the *blaZ *regulatory genes (see Tables 1 and 2).

Within the length of *blaI *region analyzed (351 nucleotides), we detected 13 unique SNP, which account for the nine *blaI *allotypes detected (see Tables 3 and 4). Four of the nine *blaI *allotypes were present in both MRSA and MSSA, while three *blaI *allotypes were found in MRSA strains only and two in MSSA only. The SID was higher for MRSA than for MSSA although not statistically significant (SID = 82.1, 95%CI 74.6-89.5 *vs *SID = 74.2, 95%CI 60.5-87.9, respectively) (Table 4). On average, each *blaI *allele has 3.4 SNP comparing to the prototype *blaI *sequence of Tn*552 *(allele 1), and *bla*I alleles were on average more polymorphic for MRSA than for MSSA (3.9 *vs *2.5 SNP per allele, respectively) - see Tables 3 and 4.

Within the length of *blaR1 *region analyzed (498 nucleotides), we detected 65 unique SNP, which account for the 12 *blaR1 *allotypes detected (see Tables 3 and 4). Six of the 12 *blaR1 *allotypes were present in both MRSA and MSSA, while four *blaR1 *allotypes were unique for MRSA strains and two were characteristic of MSSA strains. The SID values were virtually identical for both MRSA and MSSA (SID = 88.8, 95%CI 83.2-94.4 *vs *SID = 88.2, 95%CI 81.2-95.3, respectively) (Table 4). On average, each *blaR1 *allele has 24.8 SNP comparing to the prototype *blaR1 *sequence of Tn*552 *(allele 1), with no significant differences between MRSA and MSSA (24.4 and 24.6 SNP/allele, respectively) - see Tables 3 and 4.

**Figure 3:**
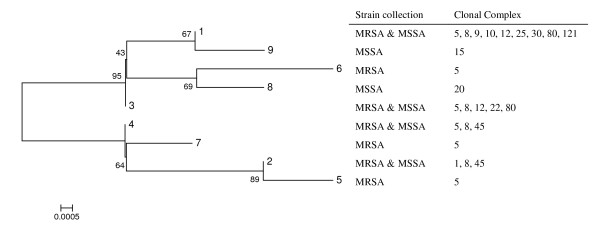
**Cluster tree of *blaI *gene allotypes found in the MRSA and MSSA collections**. See Figure 2 legend for details.

**Figure 4:**
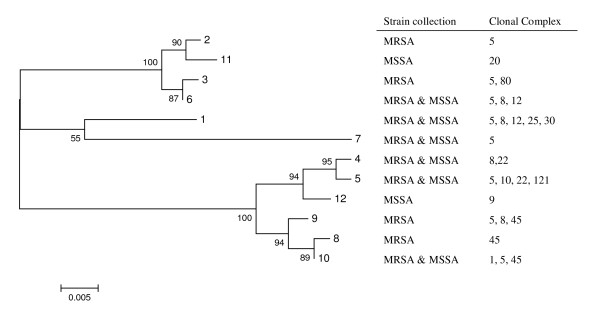
**Cluster tree of *blaR1 *allotypes found in the MRSA and MSSA collections**. See Figure 2 legend for details.

**Figure 5:**
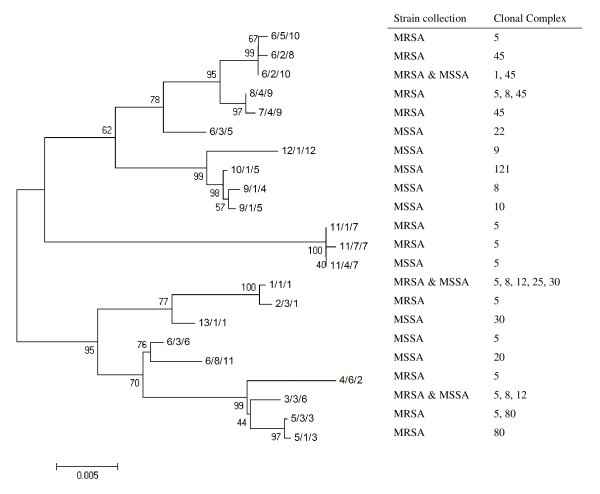
**Cluster tree of the concatenated *blaZ-blaR1-blaI *sequences found in the MRSA and MSSA collections**. See Figure 2 legend for details.

In agreement with what was observed for the *blaZ *gene, the cluster trees of *blaI *and *blaR1 *alleles found in our collections also showed no clustering according to MSSA/MRSA phenotype or genetic lineages (Figures 3 and 4). For those strains in which the alleles of the three genes were determined, we constructed a cluster tree with the concatenated sequences - see Figure 5. In spite of the relatively low number of allelic profiles, there was still no clear clustering of *bla *allotypes according to MSSA/MRSA phenotype or lineage, as the same allelic profile was present in different genetic lineages (e.g. profile 8/4/9 present in clonal complexes 5, 8 and 45) and, the same genetic lineage was characterized by profiles from different brunches (e.g. clonal cluster 8 characterized by profiles 8/4/9, 1/1/1, 3/3/6, etc.).

The BlaI and BlaR1 variabilities at the protein level in the MRSA and MSSA strains were evaluated by comparison of the deduced amino acid sequence of all alleles against the corresponding deduced amino acid sequences of Tn*552 *(see Tables 3 and 4). Overall, the deduced amino acid sequences of the *blaI *alleles revealed on average 2.3 silent mutations, 0.1 conservative missense mutations and 1 non-conservative missense mutation per allotype. The deduced amino acid sequences of the *blaR1 *alleles showed on average 10.2 silent mutations, 5.3 conservative missense mutations and 8.1 non-conservative missense mutations per allotype. None of the SNP detected within the *blaI *or *blaR1 *resulted in nonsense or frameshift mutations.

### Selection pressure acting on the *bla *locus

Based on the allelic data obtained, we computed the dN/dS ratios as estimates for the selective pressure acting on the *bla *locus. The dN/dS ratios were computed for all pairs of alleles differing more than 1%, in order to give an estimate of the allelic divergence, excluding the anomalous dN/dS ratios of those pairs being very similar. The average of the obtained dN/dS values and respective standard deviations are summarized in Table 4. The dN/dS values for the three genes in the MRSA, MSSA and MRSA/MSSA partitions were well below 1 (between 0.08 and 0.25 with standard deviations between 0.05 and 0.1), which suggests a negative or purifying selection acting on the *bla *locus. In agreement with the average number of SNP per allele, the dN/dS ratios were significantly higher for the *blaR1 *gene (0.24 - 0.25) and lower for *mecI *(0.08 - 0.11).

## Discussion

The rationale for this study comes from several observations strongly suggesting a role of *bla *genes in the acquisition, stabilization and regulation of *mecA *gene, the central element of "broad-spectrum" β-lactam resistance characteristic of MRSA strains. The purpose of this study was to evaluate the allelic variability of the *bla *locus in a representative collection of international epidemic MRSA clones and also, for comparative purposes, in a diverse collection of MSSA strains, in an attempt to establish evolutionary correlations between *bla *allotypes and β-lactam resistance phenotypes (i.e. between MRSA and MSSA), SCC*mec *types (i.e. polymorphisms in the *mecA *regulatory locus) and/or genetic lineages.

MRSA lineages are much less diverse than MSSA lineages in terms of their genome content, a consequence of their more recent evolutionary history [19,20] and, apparently, also due to some "host barrier" to the SCC*mec *acquisition [13]. These differences in genetic background variability were well illustrated in our collections since the international MRSA collection comprised eight lineages as defined by MLST clonal complexes, whereas in the smaller and local MSSA collection 15 lineages were represented.

In contrast to the genetic background diversity, we could not detect significant differences between MSSA and MRSA in terms of the *bla *locus allelic variability. Actually, there were disparate subtle differences in terms of number of allotypes and number of point mutations per allotype: e.g. 11 *vs *9 *blaZ *allotypes and 11.4 *vs *14.7 SNP/allele in MRSA and MSSA, respectively. These subtle differences may reflect the more ancient evolutionary history of MSSA or a selective pressure to improve the *bla *locus activity in these strains. That is to say, although fewer *bla *types have been retained by the natural selection in MSSA, on average, these allotypes seem to have accumulated more adaptive mutations, in comparison to MRSA strains. In particular for *blaZ*, for which differences in terms of number of alleles and SNP/allele were more significant, the presence of the alternative β-lactam resistance mechanism mediated by the *mecA *gene in MRSA strains might have allowed a release in the selective pressure to keep *blaZ *with optimal activity, in contrast to MSSA, which rely only on *blaZ*-mediated resistance to β-lactams.

No correlation could be established between *bla *allotypes and strain backgrounds, β-lactam resistance phenotypes, strain origin and/or isolation dates, indicating that *bla *genes have evolved independently from *S. aureus *clonal lineages. This is particularly striking for MRSA strains, which have a very strong clonal structure. These observations may be explained either by differences in evolutionary clock speeds between the genetic background and the *bla *locus or may result from the horizontal transfer of *bla *genes between different lineages, which are usually integrated in mobile elements (plasmids and composite transposons). Interestingly, based on the characterization of a collection of several staphylococcal species, Olsen *et al*, suggested that there is little exchange of *bla *genes between strains or species [14], which somehow contradicts our findings. In our study, the most parsimony explanation for the presence of the same *bla *type in different genetic lineages either MRSA or MSSA or the presence of several *bla *types in the same lineage, is indeed a high frequency for the horizontal transfer of *bla *genes across *S. aureus *clonal clusters.

In spite of the lack of evolutionary links between *bla *allotypes and genetic lineages, our data strongly suggests a selective pressure to keep the *bla *locus fully functional, as illustrated by the calculated average dN/dS values well below 1. This observation is valid even on MRSA for which one could expect the accumulation of nonsense or frameshift mutations that would render the *bla *locus non-functional, due to presence of the *mecA *gene. Actually, the majority of the mutational events detected in this study were either silent or neutral mutations, being the *blaR1 *the gene with the highest mutational rate and the *blaI *the one with the lowest. The increased allelic variability detected for *blaR1 *(in terms of number of alleles, Simpson's index of diversity, average SNP/allele, and dN/dS values) may suggest that this sensor-inducer gene is the primary target for the evolutionary adaptive mechanisms in the *bla *locus, presumably to improve the induction efficiency of *blaZ *expression or even *mecA *expression, in the case of MRSA strains with no functional *mecI-mecR1 *regulatory system. In contrast, the relatively lower variability of the much smaller *blaI *gene, may suggest a fine-tuned repressor activity and a selective pressure to maintain the repressor activity; i.e to maintain the *blaZ *expression inducible.

Despite the cross-resistance to virtually all β-lactam antibiotics provided by *mecA*, most contemporary MRSA strains still carry, besides the SCC*mec *element, the β-lactamase locus. This might be due to the fact that not enough time has elapsed since the *mecA *acquisition for MRSA strains start loosing the *bla *genes, because there is a little or no fitness cost associated to the *bla *genes, or because these genes may be linked to other positively selected genes (e.g. the cadmium resistance genes present in some β-lactamase plasmids). Alternatively, the *bla *locus may be involved in the "domestication" of the *mecA *gene, as *bla *genes have been shown to stabilize the *in vitro mecA *acquisition [12,13] and efficiently control *mecA *transcription [9,10], explaining the "retention" of a functional *bla *regulatory system by most contemporary MRSA strains [8]. Interestingly, as no correlation could be established between *bla *allotypes and SCC*mec *types, which have polymorphisms in the *mecA *regulatory locus, this maintenance of functional *blaI-blaR1 *genes seems to be independent of the functional status of the *mecA *"natural" regulators *mecI-mecR1*.

Concerning the maintenance of a functional *blaZ *gene in MRSA strains one can speculate that, even in the presence of *mecA*, it might be useful for the bacteria to keep *blaZ *as a "first-line defense" against β-lactams. In fact, first generation β-lactams (i.e. penicillins) are still widely prescribed either empirically or for the treatment of specific infections (e.g. streptococcal infections). Moreover, penicillins have also been widely used prophylactically in the livestock industry. This means that, both in the nosocomial and community settings, MRSA are still exposed to penicillins and, under these circumstances, expression of β-lactamase is enough for survival under antibiotic pressure. From a physiological perspective, this ability to choose between the expression of two resistance genes may be advantageous for the bacteria since the expression of β-lactamase is likely to impose a smaller fitness cost than the expression of PBP2a. In fact, besides being much smaller than PBP2a (257 *vs *668 amino acids), BlaZ is a secreted enzyme whereas PBP2a is a transpeptidase protein, which must be incorporated into the complex cell-wall metabolism.

## Conclusion

In this study we have evaluated the allelic variation of the *bla *locus in MRSA and MSSA clinical strains. Although no correlation between *bla *allotypes and genetic lineages, SCC*mec *types and β-lactam resistance phenotypes could be established, we provided evidence for the existence of a selective pressure to maintain the *bla *system fully functional even on MRSA strains and that the sensor-inducer gene *blaR1 *is the primary target for the accumulation of adaptive mutations in the *bla *locus.

## Competing interests

The authors declare that they have no competing interests.

## Authors' contributions

CM participated in the study design, carried out experimental work, analyzed and interpreted data and wrote the manuscript. AP carried out experimental work and analyzed data. LK analyzed and interpreted data. HdL participated in study design and corrected the manuscript. DCO conceived the study, participated in the study design, interpreted the data and wrote the manuscript. All authors have read and approved the manuscript.
